# Non-Tumor CCAAT/Enhancer-Binding Protein Delta Potentiates Tumor Cell Extravasation and Pancreatic Cancer Metastasis Formation

**DOI:** 10.3390/biom11081079

**Published:** 2021-07-22

**Authors:** JanWillem Duitman, Leonie Hartl, Joris J. T. H. Roelofs, Maarten F. Bijlsma, C. Arnold Spek

**Affiliations:** 1 Laboratory for Experimental Oncology and Radiobiology, Center for Experimental and Molecular Medicine, Amsterdam UMC, University of Amsterdam, 1012 VT Amsterdam, The Netherlands; J.W.Duitman@amsterdamumc.nl (J.D.); m.f.bijlsma@amsterdamumc.nl (M.F.B.); C.A.Spek@amsterdamumc.nl (C.A.S.); 2 Cancer Center Amsterdam, Amsterdam UMC, University of Amsterdam, 1012 VT Amsterdam, The Netherlands; 3 Department of Pathology, Amsterdam UMC, University of Amsterdam, 1012 VT Amsterdam, The Netherlands; J.J.Roelofs@amsterdamumc.nl; 4 Oncode Institute, 3521 AL Utrecht, The Netherlands

**Keywords:** CCAAT/enhancer-binding protein delta, CEBPD, pancreatic cancer, PDAC, extravasation, metastasis

## Abstract

CCAAT/enhancer-binding protein delta (C/EBPδ) is a transcription factor involved in apoptosis and proliferation, which is downregulated in pancreatic ductal adenocarcinoma (PDAC) cells. Loss of nuclear C/EBPδ in PDAC cells is associated with decreased patient survival and pro-tumorigenic properties in vitro. Interestingly however, next to C/EBPδ expression in tumor cells, C/EBPδ is also expressed by cells constituting the tumor microenvironment and by cells comprising the organs and parenchyma. However, the functional relevance of systemic C/EBPδ in carcinogenesis remains elusive. Here, we consequently assessed the potential importance of C/EBPδ in somatic tissues by utilizing an orthotopic pancreatic cancer model. In doing so, we show that genetic ablation of C/EBPδ does not significantly affect primary tumor growth but has a strong impact on metastases; wildtype mice developed metastases at multiple sites, whilst this was not the case in C/EBPδ^-/-^ mice. In line with reduced metastasis formation in C/EBPδ^-/-^ mice, C/EBPδ-deficiency also limited tumor cell dissemination in a specific extravasation model. Tumor cell extravasation was dependent on the platelet-activating factor receptor (PAFR) as a PAFR antagonist inhibited tumor cell extravasation in wildtype mice but not in C/EBPδ^-/-^ mice. Overall, we show that systemic C/EBPδ facilitates pancreatic cancer metastasis, and we suggest this is due to C/EBPδ-PAFR-dependent tumor cell extravasation.

## 1. Introduction

Pancreatic ductal adenocarcinoma (PDAC) is a deeply devastating disease with the worst survival outcome of any human cancer. The age-standardized incidence rate of 4.9 per 100,000 individuals worldwide constantly rises, resulting in 495,773 new cases with 466,003 deaths worldwide in 2020 [1]. The overall 5-year survival rate has slightly risen to 9%, with a mortality-to-incidence ratio of 94% [2]. The high mortality rate results from the majority of patients presenting with locally advanced tumors and/or metastatic disease, which is inoperable, rapidly progressing and eventually fatal. While some important progress has been made to improve survival in patients eligible for resection and to improve the quality of life for patients with unresectable disease, overall survival rises only slowly and currently available treatment options remain insufficient to cure the disease. Although combination therapies with different chemotherapeutic agents, such as FOLFIRINOX or gemcitabine/nab-paclitaxel, are superior to single-drug regimens, the survival benefit in patients eligible for this treatment, is only minimal [3,4].

CCAAT/enhancer-binding protein delta (C/EBPδ) is a member of the C/EBP family of transcription factors consisting of six members (C/EBPα-C/EBPζ) [5]. Originally, C/EBPδ was identified as a transcription factor driving the acute phase response, but soon after its discovery, it was also associated with cell proliferation. Nowadays, C/EBPδ is believed to act as a tumor suppressor due to its ability to regulate important cell cycle genes and pro-apoptotic signaling in cancer cells [6,7,8,9]. In line with these findings, downregulation of C/EBPδ due to hypermethylation is observed in various cancers and is therefore associated with metastatic relapse in breast cancer [6,10,11,12,13]. Moreover, C/EBPδ levels correlate with low-grade histology and disease-free survival in meningioma and breast cancer patients [14,15,16]. Finally, we recently demonstrated that nuclear C/EBPδ expression was lost in PDAC cells in patients and that loss of ductal tumor cell C/EBPδ correlated with lymph node involvement and overall survival in these patients [17]. In PDAC cell lines, re-expression of C/EBPδ slowed down proliferation and decreased the clonogenic capacity of PDAC cells in a dose-dependent manner, while knock-down of C/EBPδ reversed these effects.

C/EBPδ expression is not limited to tumor precursor epithelial cells. Amongst others, it is expressed in endothelial cells, macrophages and nervous tissues [18,19,20,21,22]. The (patho)physiological importance of C/EBPδ in these non-tumor cells is not particularly well understood but it is important to realize that these cell types are major constituents of the pancreatic cancer microenvironment and the site of extravasation and metastasis formation. Based on these considerations, we specifically addressed the importance of non-tumor cell C/EBPδ expression on tumor growth and the formation of metastases. Using an orthotopic PDAC model and a specific extravasation model, we show that systemic C/EBPδ facilitates metastases by promoting tumor cell extravasation.

## 2. Materials and Methods

### 2.1. Tissue Microarray (TMA)

Formerly described TMAs [17] were revised by an experienced pathologist to discern different stromal cell types.

### 2.2. Mining of Publicly Available RNA Microarray Datasets

Datasets were derived from the Gene Expression Ominbus (National Institutes of Health, Bethesda, MD, USA) [23], ArrayExpress (EMBL-EBI, Cambridge, UK; CIBIO/InBIO-Centro de Investigação em Biodiversidade e Recursos Genéticos, Vairão, PT) [24] and the Genotype-Tissue Expression (GTEx) Portal (Broad Institute of MIT and Harvard University, USA) using the R2 microarray analysis and visualization platform [25]. To examine tumor and tumor stroma *CEBPD,* mRNA expression levels were derived from two different datasets, i.e., GSE93326 and E-MEXP-1121. From the GSE93326 dataset by Renz et al. [26], which is a microdissected dataset comprised solely of samples originating from pancreatic ductal adenocarcinomas, all samples annotated as either epithelial or stromal were included. From the E-MEXP-1121 dataset by Pilasrky et al. [27], all samples derived from PDAC and annotated as either stromal or epithelial were included. From the GTEx Project, the current v8 release was used.

### 2.3. Mice

C/EBPδ^-/-^ mice, generated as previously described [22], and C57BL/6 mice (purchased from Charles River) were maintained at the animal facility of the Academic Medical Center of Amsterdam with free access to food and water. All animal experiments were approved by the Academic Medical Center’s institutional Animal Care and Use Committee. In order to detect a minimal effect size of 50% with statistical significance of 0.05 and a power of 80%, we included 8 mice per experimental group in the animal models described below.

### 2.4. Cells and Cell Culture

Panc02 (RRID: CVCL_D627, kindly provided by Dr. Schmitz, University Hospital Bonn) and B16F10 (RRID: CVCL_0159, ATCC-CCL-6475) cells were confirmed to be mycoplasma-free and maintained at 5% CO_2_ and 37 °C in RPMI 1640 or DMEM, respectively, (Invitrogen, Carlsbad, CA, USA) supplemented with 10% fetal bovine serum (FBS; Lonza, Switzerland), 1% penicillin-streptomycin and 1% L-glutamine (Lonza, Switzerland). Single cell suspensions were prepared from 2 mM EDTA-treated monolayers, which were washed and diluted in phosphate-buffered saline (PBS) prior to counting and inoculation. Cells were stored on ice until injection.

### 2.5. Orthotopic Pancreatic Cancer Model

Mice were subjected to an orthotopic model of pancreatic cancer as described previously [28,29]. Briefly, confluent cultures of Panc02 cells (>90% viable) were detached using 2 mM EDTA, and centrifuged at 800 RPM for five minutes, washed twice with phosphate-buffered saline (PBS) and resuspended in 0.9% sterile saline (Sigma, St Louis, MO, USA). Tumor cells (4.0 × 10^5^ cells per mouse) were injected directly into the pancreas. Mice were evaluated for changes in body weight and signs of discomfort or morbidity daily and were euthanized five weeks after tumor cell injection. The pancreas, including the tumor, was removed, measured, weighed and preserved in 10% formalin. Spleen, liver, mesentery and lungs were macroscopically inspected for metastases and preserved in 10% formalin afterwards.

### 2.6. Experimental Extravasation Model

B16F10 cancer cells (3.5 × 10^5^) suspended in 200 μL PBS were injected into the lateral tail vein as described previously [30,31]. After inoculation of the cancer cells, wildtype and C/EBPδ^-/-^ mice were divided into two equal groups (n = 8). One group was treated intraperitoneally (i.p.) with the PAFR antagonist WEB2086 (#2339, Tocris Bioscience, Bristol, UK) at 5 mg/kg of body weight in 3% DMSO in PBS for three consecutive days starting at day 1 as described previously [32]. The other group served as a solvent control and was therefore treated with 3% DMSO in PBS only. Fourteen days after cancer cell injection, the mice were anesthetized and sacrificed by vena cava puncture. Lungs were fixed directly with 4% paraformaldehyde administered through the trachea and were removed afterwards. The lungs were kept in formaldehyde solution until substituted for 70% alcohol after 24 h. Tumor foci on the surface of the lungs were counted macroscopically in a blinded fashion with respect to the intervention.

### 2.7. Data Analysis

Statistical analyses were performed using GraphPad Prism 6.0 (GraphPad Software Inc., La Jolla, CA, USA). Data were analyzed for normality using the Shapiro–Wilk test. Following this, parametric or non-parametric tests were applied accordingly. *CEBPD* expression in tumor epithelium and tumor stroma was analyzed using the Mann–Whitney U test, the tumor weight of WT and C/EBPδ^-/-^ animals was compared using an unpaired *t*-test, the frequency of metastasis formation in individual organs was compared between WT and C/EBPδ^-/-^ animals using Fisher’s exact test and finally, to compare metastasis formation upon PAFR-inhibition in both treatment arms of the WT or C/EBPδ^-/-^ group, the Mann–Whitney U test or the unpaired t-test was used.

## 3. Results

### 3.1. C/EBPδ Is Not Tumor-Specific and Is Widely Expressed in Different Tissues

**Figure 1:**
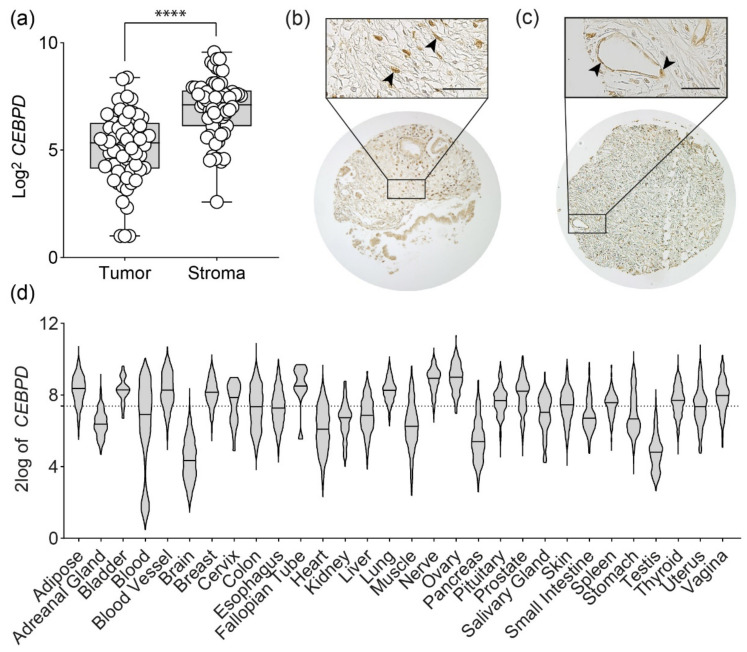
C/EBPδ expression is evident in the stromal compartment of PDAC: (**a**) mRNA expression of *CEBPD* is significantly enhanced in the stromal compartment compared to epithelial tumor cells in the publicly available microdissected mRNA dataset by Renz et al. (**** *p* < 0.0001, Mann–Whitney U test) [26]. (**b**,**c**) Immunohistochemical stainings for C/EBPδ in PDAC biopsies show strong C/EBPδ protein expression in stromal cells such as fibroblasts (**b**) and endothelial cells (**c**). Black arrowheads indicate fibroblasts and endothelial cells stained positive for C/EBPδ. Scale bar is 40 µm. Upper panels are imaged at 40× magnification, lower panels are imaged at 10×. (**d**) *CEBPD* mRNA is also widely expressed throughout different tissues of the body as shown here based on data derived from the Genotype-Tissue Expression (GTEx) Project. Black vertical lines in violin plots show the respective mean and the dotted black line at y = 7.38 shows the overall median of all shown datasets.

In our previous work on the role of C/EBPδ in PDAC cells, we found that C/EBPδ protein and CEBPD mRNA are expressed in the ductal cells of a healthy pancreas but lost in PDAC cells, while re-expression of C/EBPδ drastically reduced PDAC cell tumorigenicity [17]. In this study, we set out to investigate the effects of non-tumor C/EBPδ on pancreatic cancer growth and metastatic disease. To this end, we first looked at the site of the tumor and examined two publicly available microdissected mRNA datasets to investigate the expression of *CEBPD* mRNA in the tumor and tumor stroma. As exemplarily shown in the data of Renz et al. [26], we indeed found that *CEBPD* mRNA expression was significantly enhanced in the stromal compartment as compared to the epithelial compartment of pancreatic ductal adenocarcinomas (Figure 1a, *p* < 0.0001; data accessible at Gene Expression Omnibus [23], accession GSE93326). Similar results were obtained from the Pilarsky et al. dataset [27] (*p* = 0.0019; data accessible at ArrayExpress [24], accession E-MEXP-1121), implying that C/EBPδ indeed plays an active role in these tissues. Interestingly, this difference in *CEPBD* mRNA expression between tumor and stromal cells seems not to be a general phenomenon. *CEBPD* is often hyper-methylated and is associated with good prognosis in breast cancer [10,15] and has tumor-suppressive functions in ovarian cancer [30]. Only in ovarian cancer do we find a significant difference between tumor cell *CEBPD* and stromal *CEBPD* (*p* < 0.0025). No significant difference in *CEBPD* mRNA expression was observed between tumor epithelia and stromal cells in a much larger breast cancer dataset (Supplementary Figure S1) [31,32]. Yet, we again found that *CEBPD* mRNA was widely expressed in the tumor stroma. Given that primarily nuclear C/EBPδ is considered active and biologically relevant, we next revised previously performed stainings of C/EBPδ in in-house made PDAC microarrays [17]. We found that C/EBPδ protein was not only strongly expressed in nuclei of normal epithelial cells but also in nuclei of fibroblasts (Figure 1b) and endothelial cells (Figure 1c), while C/EBPδ was almost completely absent in tumor cell nuclei. Moving away from the tumor into other tissues of the body, along with potential metastatic sites, we next assessed *CEBPD* mRNA expression in healthy tissue using the Genotype-Tissue Expression (GTEx) Project dataset. Not surprisingly, this revealed that *CEBPD* was expressed in most tissues of the body (Figure 1d). This emphasizes that non-tumor C/EBPδ may play a role in various biological processes, including carcinogenesis, and prompted us to investigate the effect of host C/EBPδ knock-out on tumor growth and the formation of distant metastases.

### 3.2. Non-Tumor C/EBPδ Potentiates Pancreatic Cancer Metastases

**Figure 2:**
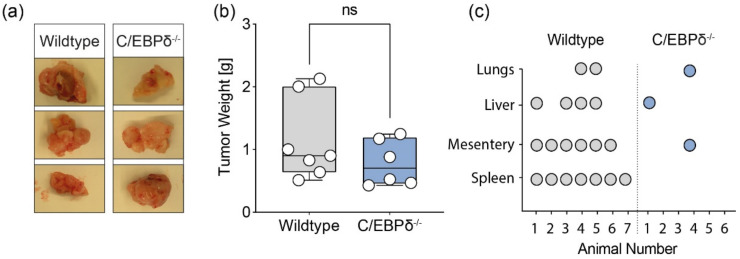
Stromal C/EBPδ potentiates metastasis without affecting primary PDAC growth: (**a**) representative macroscopic images of pancreatic tumors derived from wildtype or C/EBPδ^-/-^ mice orthotopically injected with C/EBPδ-proficient pancreatic tumor cells. Tumors were resected 5 weeks after injection and show no difference in either volume or weight between wildtype and C/EBPδ^-/-^ host animals. (**b**) Weight of orthotopically injected pancreatic tumors upon resection. Shown is the mean +/- SEM; An unpaired t-test was applied; ns: not significant. (**c**) Location of metastasis in individual animals in the wildtype (n = 7) and C/EBPδ^-/-^ (n = 6) groups. Fisher’s exact tests yield *p* < 0.005 and *p* < 0.05 between wildtype and C/EBPδ^-/-^ spleen and mesentery, respectively; p-values are not significant between wildtype and C/EBPδ^-/-^ lungs and liver.

To obtain proof of principle for the potential role of the host’s C/EBPδ expression in pancreatic cancer and metastatic disease, wildtype and C/EBPδ^-/-^ mice were subjected to an orthotopic pancreatic cancer model where C/EBPδ proficient murine pancreatic cancer cells were orthotopically injected into the pancreas. Primary tumor growth, as determined by weight of the tumor at sacrifice four weeks after cancer cell injection, was slightly but not significantly reduced in C/EBPδ^-/-^ mice as compared to wildtype mice (Figure 2a,b) (weight of 1.15 ± 0.25 vs. 0.79 ± 0.15 g (*p* = 0.36) for wildtype and C/EBPδ^-/-^ mice, respectively). Accordingly, tumor volume was not significantly different between the two groups (1.29 ± 0.32 vs. 0.77 ± 0.16 cm^3^ (*p* = 0.37), data not in graph). Interestingly, however, metastases were observed in numerous organs of the grafted wildtype animals but not in C/EBPδ^-/-^ mice (Figure 2c). C/EBPδ expression by the host thus appears to support pancreatic cancer metastasis.

### 3.3. C/EBPδ Enhances Experimental Cancer Cell Extravasation

**Figure 3:**
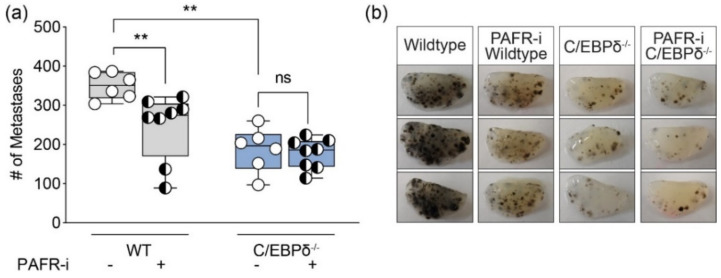
C/EBPδ potentiates tumor cell extravasation in a PAFR-dependent manner: (**a**) wildtype and C/EBPδ^-/-^ mice treated or not treated with a specific PAFR antagonist (PAF-i, WEB2086; once daily during the first three days) were injected with 3.5 × 10^5^ B16F10 tumor cells into the lateral tail vein. After 14 days, the mice were sacrificed under anesthesia, after which the number of tumor foci at the surface of the lungs was determined. Shown is the mean ± SEM; ** *p* < 0.005; ns: not significant. The Mann–Whitney U test was used to compare PAFR-i with controls in the WT group (not normally distributed) and the unpaired t-test was used to compare PAFR-i with controls in the C/EBPδ^-/-^ group, as well as base-line metastasis formation between the WT and C/EBPδ^-/-^ group without PAFR-i. (**b**) Representative lungs of wildtype or C/EBPδ^-/-^ mice with or without WEB2086-treatment. Dark dots in the lungs are melanoma tumor nodules.

To assess whether C/EBPδ could potentially drive metastases by enhancing tumor cell extravasation, we subjected wildtype and C/EBPδ^-/-^ mice to a well-established experimental extravasation model [33,34]. This model uses murine B16F10 cells and specifically measures the ability of tumor cells to extravasate in the lungs. B16F10 cells are used, as they express high levels of melanin and can thus be readily and reliably counted upon forming nodules. As shown in Figure 3, the injection of tumor cells into wildtype mice indeed efficiently led to tumor cell extravasation, as evident from the large number (n = 350 ± 14) of tumor nodules in the lungs at the time of sacrifice. Interestingly, the number of tumor nodules in the lungs of C/EBPδ^-/-^ mice (n = 186 ± 23; *p* = 0.0001 compared to wildtype mice) was dramatically reduced, suggesting that the host’s C/EBPδ expression indeed potentiates tumor cell extravasation.

Interestingly, C/EBPδ induces PAFR expression during infectious disease, whereas in a tumor setting, PAFR expression enhances tumor cell extravasation [35,36]. Consequently, we assessed whether the increased number of tumor cells extravasating in wildtype animals, as compared to C/EBPδ^-/-^ animals, may depend on differential PAFR expression. To this end, wildtype and C/EBPδ^-/-^ mice were subjected to the extravasation model in the absence or presence of a specific PAFR antagonist. As shown in Figure 3, administration of the PAFR antagonist inhibited tumor cell extravasation in wildtype but not in C/EBPδ^-/-^ mice. Host C/EBPδ thus seems to modify tumor cell extravasation in a PAFR-dependent manner.

## 4. Discussion

In the current study, we aimed to elucidate the importance of non-tumor C/EBPδ on PDAC progression. We confirm that C/EBPδ is widely expressed in the stromal compartment of human PDAC and present evidence of its expression throughout various tissues of the body, substantiating the notion that stromal C/EBPδ is not just an accompanying effect of tumor growth. Although non-tumor C/EBPδ does not affect primary pancreatic cancer growth, it significantly potentiates metastasis. The fact that metastasis is the major cause of morbidity and mortality in PDAC patients underscores the potential importance of our findings and suggests that systemic C/EBPδ is a key factor in PDAC progression.

The role of C/EBPδ in the setting of cancer research has mainly focused on C/EBPδ expression by tumor cells, and most of these studies show that C/EBPδ acts as a tumor suppressor. Indeed, C/EBPδ expression is downregulated in primary breast cancer, which correlates with metastasis and progression-free survival [15,16]. Moreover, C/EBPδ is downregulated in acute myeloid leukemia, cervical cancer and hepatocellular carcinoma, whereas epithelial C/EBPδ expression is also lost in PDAC patients [12,13,17]. In these latter patients, loss of tumor cell C/EBPδ correlated with lymph node metastasis and overall survival. In contrast to the proposed tumor suppressor role of C/EBPδ, some recent studies show that C/EBPδ over-expression in tumor cells correlates with poor prognosis in glioblastoma and with metastatic disease and reduced disease-specific survival in urothelial carcinoma [37,38]. Overall, a picture thus emerges that the role of C/EBPδ in cancer is not as straightforward as anticipated, and C/EBPδ may act in a context-dependent manner to either potentiate or inhibit cancer progression.

Although the role of C/EBPδ in somatic tissues has not been studied extensively, it is widely assumed that, due to its function in the induction of inflammation, C/EBPδ does play a role in the inflammatory tumor microenvironment and overall stroma [39]. Indeed, stromal C/EBPδ was previously found to be activated in cancer-associated macrophages and fibroblasts, leading to enhanced metastasis, chemoresistance and stemness [40]. Here, we provide additional evidence for a context-dependent role of C/EBPδ in cancer biology by showing a metastasis-promoting effect of non-tumor C/EBPδ in the setting of PDAC. Of note, in an inflammatory context, C/EBPδ is known to impact function and extravasation of specific T cells [41]. Given the increasing recognition of the importance of the immune cell receptor repertoires in the tumor microenvironment and beyond [42], it would be interesting to study immune cell populations and T cell repertoires using our model.

In the current manuscript, we employed an orthotopic pancreatic cancer model in which murine Panc02 cells were grafted into the pancreas of immune competent wildtype or C/EBPδ^-/-^ mice. This orthotopic model yields a tissue-specific pathology and is generally considered more clinically relevant than xenograft models [43,44]. In this model, the formation of metastases was largely reduced in C/EBPδ^-/-^ mice compared to wildtype mice, while primary tumor growth remained unaffected by the status of stromal C/EBPδ. To substantiate these findings, we further analyzed C/EBPδ-dependent formation of metastases in a specific extravasation model using mouse melanoma skin cells. As opposed to PDAC cells, melanoma cells express high levels of melanin and therefore form dark nodules in the lungs which are clearly visible and allow for reliable counting and quantification. This model is well suited to the study of tumor cell extravasation in a well-timed manner without confounding variables present in primary tumor models [30,34]. We opted for such an extravasation model based on the notion that, in contrast to tumor cell intravasation, tumor cell extravasation is a rate limiting step in metastasis formation [45,46,47]. Using this approach, we found that the number of pulmonary tumor nodules in wildtype mice was increased compared to that of C/EBPδ^-/-^ mice, implying that impaired extravasation, at least in part, also accounts for the limited formation of metastases in our orthotopic PDAC model.

In the setting of infectious disease, C/EBPδ has been shown to regulate PAFR expression, thereby compromising the epithelial barrier function and exaggerating bacterial dissemination [36]. This is particularly interesting, as PAFR activation is also known to induce endothelial barrier disruption [48,49]. Moreover, platelet-activating factor (PAF), the endogenous PAFR ligand, is known to promote tumor initiation and to enhance tumor cell extravasation and metastasis formation in vivo, while this effect is inhibited by a PAF antagonist [35,50]. Here, we show that PAFR inhibition, using WEB2086, efficiently inhibits tumor cell extravasation in wildtype mice as opposed to C/EBPδ^-/-^ mice in which WEB2086 had no effect on extravasation. Importantly, however, PAFR antagonist administration in wildtype mice is not as effective as genetic ablation of C/EBPδ. This may indicate that C/EBPδ-dependent tumor cell extravasation is only partly PAFR-dependent (although sub-optimal targeting by the PAFR antagonist cannot be excluded as a possible explanation for the difference between targeting versus genetic ablation). The underlying mechanism of this dependency remains obscure and, importantly, it cannot be excluded that the PAFR antagonist also acts on the injected tumor cells to modify their extravasation potential via unexamined mechanisms. Irrespectively, we emphasize here another controversial role of C/EBPδ in the context of carcinogenesis and, specifically, in metastasis formation. Further studies are needed to clarify whether our findings can be extrapolated from PDAC and the melanoma-based extravasation model to other cancers.

## 5. Conclusions

In conclusion, we show by the example of an orthotopic PDAC model that non-tumor C/EBPδ drives metastasis formation and suggest this is due to C/EBPδ-PAFR-dependent tumor cell extravasation. These data point to a novel role of C/EBPδ in carcinogenesis and suggest that C/EBPδ plays a context-dependent role in pancreatic cancer progression by acting as a tumor suppressor in tumor cells as well as a tumor promotor in the non-tumor compartment [17].

## Data Availability

The data that support the findings of this study are openly available in Gene Expression Omnibus at https://www.ncbi.nlm.nih.gov/geo/query/acc.cgi?acc=GSE93326 (accessing date: 05 March 2019), reference number 23 and 26, in ArrayExpress at https://www.ebi.ac.uk/arrayexpress/experiments/E-MEXP-1121/ (accessing date: 20 April 2016), reference number 24 and 27 and in the GTEx Portal at https://www.gtexportal.org (accessing date: 29 February 2020). The Genotype-Tissue Expression (GTEx) Project was supported by the Common Fund of the Office of the Director of the National Institutes of Health, and by NCI, NHGRI, NHLBI, NIDA, NIMH and NINDS. Data generated during this study are available from the corresponding author upon reasonable request.

## Supplementary Materials

The following are available online at https://www.mdpi.com/article/10.3390/biom11081079/s1, Figure S1: *CEBPD* mRNA is significantly differentially expressed between tumor epithelial cells and tumor stromal cells in ovarian cancer but not in breast cancer.
