# Supplementary material for: Non-Tumor CCAAT/Enhancer-Binding Protein Delta Potentiates Tumor Cell Extravasation and Pancreatic Cancer Metastasis Formation
Source: Biomolecules. 2021 Jul 22;11(8):1079. doi: 10.3390/biom11081079 (PMC8391339; doi:10.3390/biom11081079)
Supplement: Supplementary file 1 [file biomolecules-11-01079-s001.zip › biomolecules-1291308-supplementary.pdf]

Supplementary Material

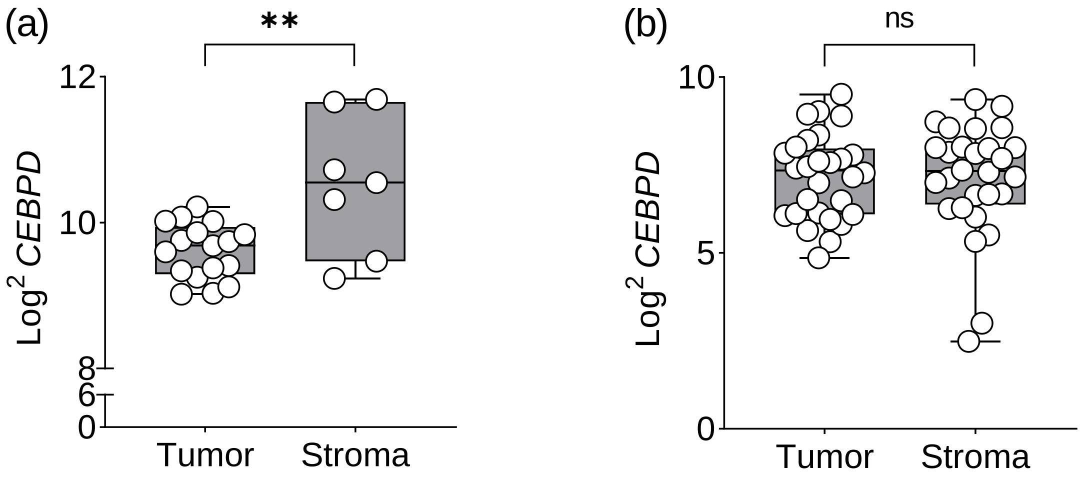

Figure S1. *CEBPD* mRNA is significantly differentially expressed between tumor epithelial cells and tumor stromal cells in ovarian cancer but not in breast cancer. (a) Data are derived from Lili et al., GSE38666. Tumor = ovarian cancer epithelium (N=17), Stroma = ovarian cancer stroma (N=7) (**p<0.0025) [31]. (b) Data are derived from Casey et al., GSE10797. Tumor = invasive breast cancer tumor epithelium (N=28), Stroma = invasive breast cancer stromal cells (N=28); ns: not significant [32].
